# Dexmedetomidine: Shifting Paradigms in Neonatal Sedation and Pain Control

**DOI:** 10.3390/children12040444

**Published:** 2025-03-30

**Authors:** Kok Joo Chan, Srinivas Bolisetty

**Affiliations:** 1 Newborn Care Centre, Royal Hospital for Women, Sydney, NSW 2031, Australia; chankj@ummc.edu.my; 2 Discipline of Paediatrics & Child Health, University of New South Wales, Sydney, NSW 2031, Australia; 3 Neonatology Unit, Department of Pediatrics, University of Malaya Medical Centre, Kuala Lumpur 59100, Malaysia; 4 Medical Co-Director of Newborn Care, Royal Hospital for Women, Sydney, NSW 2031, Australia

**Keywords:** sedation, prematurity, low birth-weight

## Abstract

Background: Newborns, including preterm infants, are capable of responding to pain. Recurrent pain exposure is associated with suboptimal motor development, cognitive impairments, abnormal brain growth, and maladapted nociceptive reactions. Problem: Current agents, primarily opioids and benzodiazepines, raise major concerns due to their adverse effects, including insufficient sedation or analgesia, withdrawal, depressed respiratory effort, tolerance, and occasional paradoxical agitation. Commonly used drugs such as midazolam and morphine have been shown to induce neuroapoptosis and neurodevelopmental abnormalities in animal studies. Evaluation—Dexmedetomidine: As a specific alpha-2 adrenergic agonist, dexmedetomidine causes a significantly lower reduction in breathing effort. It has over 800 times greater affinity for alpha-2 receptors compared to alpha-1 receptors. Common side effects include bradycardia and hypotension. Prolonged use may necessitate a transition to clonidine during the weaning process. Dexmedetomidine can be administered intravenously as a bolus or infusion or intranasally. Indications include sedation and analgesia for mechanical ventilation, therapeutic hypothermia, procedural premedication, and as an adjunct to inhalational anesthesia and nerve-blocking agents. Research across varying age groups has demonstrated that dexmedetomidine shortens periods of invasive ventilation and decreases the need for other sedatives. Neonatal studies suggest that dexmedetomidine may help accelerate the achievement of full enteral feeds and can be safely administered within specific dosage ranges without causing significant adverse events that would necessitate abrupt discontinuation. Conclusions: Dexmedetomidine can be used alone or in combination with other agents. By increasing the use of dexmedetomidine, it is possible to reduce the dosage of concurrent medications, thereby minimizing the risk of complications while still achieving the desired sedation and analgesia.

## 1. Current Conundrum

Currently, neonatal sedation and analgesia are primarily achieved using opioids, especially for invasive ventilation and procedural or surgical pain control. European Pain Audit In Neonates (EUROPAIN), a prospective cohort study conducted in neonatal intensive care units (NICUs) across European nations, reported that opioids were administered to 26% of patients admitted to NICUs. Up to 74% of infants receiving tracheal ventilation were given opioids [1]. Morphine and fentanyl are generally the preferred choices for sedation during endotracheal intubation, maintenance of mechanical ventilation, and post-operative pain control [2].

Apart from the immediate to medium-term adverse effects of opioids, such as reduced breathing effort, hypotension, bradycardia, ileus, and urinary retention, there have been growing concerns about longer-term complications and potential neurodevelopmental issues [3]. While limited literature suggests that lower doses of opioids, such as continuous morphine at 10 mcg/kg/h, may be neuroprotective by conferring positive effects on executive functioning skills and daily living in children aged 8 and 9 years [4], this potential benefit may be attributed to the mitigation of pain exposure, with evidence supporting impaired brain development in preterm newborns [5]. However, with continuous morphine dosages ranging from 5 to 40 mcg/kg/h [6] and a lack of sufficient literature on the safety regarding duration and dosage, as well as varying inter-unit practices, there are reasonable concerns about opioid-related complications in the long term. Concerns arise from studies of morphine-induced neuronal apoptosis in both animal [7] and human studies [8], with potential problems including longer choice response latencies, increased parent-reported social problems, and reduced task completion rates [9].

Aside from opioids, another commonly prescribed agent is midazolam, which acts by amplifying the natural inhibitory pathways influenced by gamma-aminobutyric acid (GABA) through binding to benzodiazepine receptors [10]. This provides desirable effects such as muscle relaxation, anti-epileptic activity, and anxiolysis [11]. Midazolam may have been administered in up to 9% of total NICU admissions [1]. Animal studies have raised concerns about apoptotic neurodegeneration in the infant mouse brain with relatively mild exposure to midazolam [12]. Additional concerns include the potentially delayed clearance of midazolam metabolites due to the limited capacity of the cytochrome P450 3A4 enzyme in the livers of preterm infants, which may differ from term infants and older age groups [13]. These factors contribute to reasonable uncertainty regarding the safety of midazolam. Long-term outcome studies include a study on 138 neonates exposed to midazolam, which involved imaging with diffusion tensor scans and magnetic resonance imaging while the patients were younger and at term-equivalent age, followed by developmental assessments at around 18 months of age. This study demonstrated possible dose-dependent abnormal hippocampal growth and lower cognitive scores [14].

To balance the risks and benefits of these agents, multimodal approaches to ensure infant comfort are routinely pursued. Approaches that do not rely on medications, such as swaddling or non-nutritive sucking [15], should be considered whenever feasible. Other pharmacological adjuncts, such as paracetamol, could also be considered [16]. Nonetheless, when these efforts are insufficient and opioids and benzodiazepines are required, there is a continued need to explore safer pharmacological strategies for providing adequate sedation and pain control, particularly for sicker infants who require prolonged ventilation or major surgeries, while minimizing the risk of complications. This could be achieved by adding other agents to limit the dosages of opioids and benzodiazepines required while ensuring adequate comfort care for infants.

This narrative review aims to discuss the emerging role of dexmedetomidine in neonatal analgesia and sedation, highlighting its unique qualities and the necessary precautions in clinical applications.

## 2. New Horizon: Dexmedetomidine

**Figure 1:**
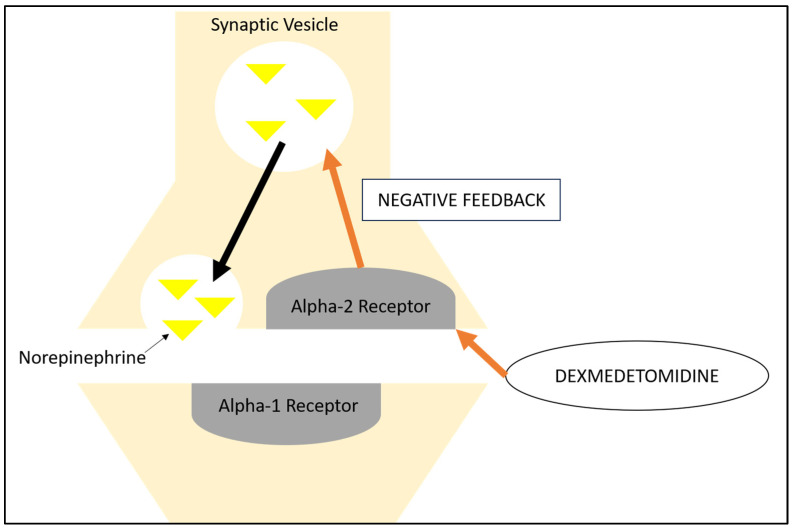
Adrenoceptor agonist receptor.

As a centrally acting alpha-2-adrenoceptor agonist, the properties of dexmedetomidine include sedation, pain control, sympatholytic effects, and anxiolysis (Figure 1). A significant proportion of dexmedetomidine is bound to proteins, with reports suggesting that up to 94% of the drug binds to alpha-1-glycoprotein and albumin [17]. Dexmedetomidine undergoes near-complete metabolism by the liver via glucuronidation, with pathways involving uridine diphosphate glucuronosyltransferase (UGT) and cytochrome P450 (CYP) enzyme 2A6, notably UGT1A4 and UGT2B10, to form inactive metabolites [18]. Renal impairment does not appear to significantly affect dexmedetomidine pharmacokinetics [17]. At the time of writing this article, the authors found no literature advocating contraindications or dose adjustments in children with liver impairment. Overall, around 1% or less is excreted unchanged. The vast majority (up to 95%) of metabolites are excreted via urine, with the remainder excreted via stool.

After the initial experiences with clonidine and the expansion of indications for alpha-2-adrenoceptor agonist drugs, the Food and Drug Administration (FDA) approved dexmedetomidine in 1999. Initially, dexmedetomidine was indicated for pain control and sedation for short durations and was primarily utilized in adult intensive care units [19]. Over the past two decades, its indications have expanded to include sedation for patients who are not invasively ventilated during procedures and surgeries, with increasing clinical applications due to its favorable physiological effects [20]. In contrast to opioids and benzodiazepines, respiratory depression is minimal with dexmedetomidine. It induces a state resembling natural sleep, with patients remaining rousable while under sedation [21], a phenomenon referred to as “arousable sedation” or “cooperative sedation” in adults [20]. Oral dryness, a frequent adverse effect of alpha-2 agonists [22], may be beneficial in certain patients as an antisialagogue [20].

In children, dexmedetomidine is commonly used concurrently with anesthetic agents in operating theaters for procedures and surgeries. It was subsequently included in sedation regimens in the pediatric intensive care unit (PICU), with the most common usage being sedation for radiological procedures [23]. In addition to intravenous administration, other routes such as oral [24], buccal [25], and intranasal [26] have expanded the potential for dexmedetomidine to be used either as a sole agent or as an adjunct to multimodal strategies. Literature comparing the efficacy of other agents to dexmedetomidine is growing. For morphine and fentanyl, a meta-analysis of five randomized controlled trials (RCTs) concluded that dexmedetomidine was equally efficacious as opioids in reducing pain and emergence agitation after adenoidectomy or tonsillectomy in the pediatric population [27]. One of the RCTs reported that dexmedetomidine produced less respiratory depression but with lower analgesic effectiveness compared to morphine [28]. Regarding benzodiazepines, a meta-analysis of eleven RCTs on pediatric pre-medication options compared midazolam to dexmedetomidine and found that dexmedetomidine may be superior in providing adequate sedation after parent–child separation and better mask compliance. Additional benefits included a reduced need for breakthrough analgesia, less delirium with agitation, and fewer shivering episodes after surgery [29]. Other purported benefits included an RCT showing that prophylactic use of dexmedetomidine may decrease junctional ectopic tachycardia after congenital heart surgery [30].

With the increasing recognition of the qualities of dexmedetomidine, it is unsurprising that it was introduced to neonatal management over the past decade. The growing trend of dexmedetomidine usage in neonates can be illustrated by a multicenter, observational cohort study that included 395,122 babies with gestational ages ranging from 22 weeks to 36 weeks in the United States from 2010 to 2020. The median birth weight was 2040 g (IQR 1606–2440 g), with a corresponding median gestational age of 34 weeks (IQR 32–35 weeks). Of this cohort, 0.1% of the infants (n = 384) received dexmedetomidine. These infants were more preterm, had lower birth weights, required prolonged intensive care stays, had increased utilization of opioids, and had longer durations of invasive ventilation. The first exposure to dexmedetomidine occurred at a median postmenstrual age of 31 weeks (IQR 27–36 weeks), with a median postnatal age of 3 days (IQR 1–35 days). Overall, patients received dexmedetomidine for a median duration of 6 days (IQR 2–14 days). In 2020, the usage of dexmedetomidine increased to 0.185% from 0.003% in 2010, which was statistically significant (*p* < 0.001). This occurred alongside a reduction in opioid exposure, with opioid utilization decreasing to 7.2% in 2020 from 8.5% in 2010, a change that was also statistically significant (*p* < 0.001). Therefore, this study highlights the incremental usage of dexmedetomidine and the reduction in opioid utilization in the preterm cohort from 2010 to 2020 [31].

For the evaluation of neonates requiring invasive ventilation, although a 2024 Cochrane review did not identify any randomized controlled trials in neonates [32], a meta-analysis and systematic review conducted by Portelli et al., 2014, included six studies involving 252 neonates [33]. These studies comprised a pair of retrospective cohorts and two pharmacokinetic studies, along with a single randomized controlled trial and a case-control study. The studies compared dexmedetomidine to either morphine, fentanyl, or placebo as a control. The focus was on sedation and potential analgesic effects for neonates receiving mechanical ventilation or undergoing therapeutic hypothermia for hypoxic-ischemic encephalopathy (HIE). Dexmedetomidine was administered intravenously in these studies. The loading dose ranged from 0.05 to 0.5 mcg/kg, with the infusion administered over 10 to 60 min. The mean maintenance infusion rate ranged from 0.05 to 1.2 mcg/kg/h. The average duration of dexmedetomidine administration ranged from 6 h to 12 days. One study focused on HIE patients receiving therapeutic hypothermia with the goal of minimizing shivering episodes. Another study analyzed the safety, effectiveness, and pharmacokinetics of dexmedetomidine in term and preterm cohorts. The review concluded that dexmedetomidine could be effective in the following cases: (1) providing analgesia and sedation, (2) minimizing the need for other medications, (3) reducing the time to extubate and shortening invasive ventilation requirements, and (4) accelerating the attainment of enteral feeding. As no major complications were reported, the review summarized that dexmedetomidine could be safely administered with appropriate neonatal dosing, avoiding sudden cessation due to medication-related complications. The caveat for appraising this review lies in the inherent bias risks associated with retrospective and non-randomized studies.

Babies with HIE requiring therapeutic hypothermia represent a unique population in neonatology, with the added imperative to minimize long-term neurological complications from all factors, including medications. An open-label RCT of dexmedetomidine in 205 term babies with HIE receiving therapeutic hypothermia in an NICU in Ukraine found that dexmedetomidine was safe and that hemodynamic stability was achievable. No negative cerebral events were reported with dexmedetomidine, and neuroprotective benefits were even postulated for this cohort, in addition to therapeutic hypothermia. A significant difference in the days of tracheal extubation (*p* = 0.022) was observed; babies in the dexmedetomidine group were significantly more likely to be extubated before 7 days of treatment, with 68% in the dexmedetomidine group compared to 33% in the control group (*p* = 0.018, HR 0.48, 95% CI 0.27–0.86, *p* = 0.011). Additionally, the near-infrared spectroscopy index rScO2 differed significantly between the groups on the first and second days of treatment: 65% to 79% for the dexmedetomidine group (*p* = 0.012) compared to 74% to 81% for the control group (*p* = 0.035). Blood pressure stability was reflected by a better mean arterial pressure in the dexmedetomidine group, which was 58 mmHg (51–65 mmHg) compared to 53 mmHg (46–60 mmHg) in the control group (*p* < 0.001). As expected, there was a lower requirement for inotropes, with reduced dobutamine doses in the dexmedetomidine group (EV −1.87, 95% CI from −3.25 to −0.48, *p* = 0.009). The frequency of seizures was significantly lower on the first day of observation in the dexmedetomidine group, with a rate of 4.3% compared to 48.3% in the control group (*p* < 0.001). Negative neurological consequences, such as cerebral leukomalacia, were approximately seven times less frequent in the dexmedetomidine group (2.2%) compared to the control group (15.1%) (*p* = 0.018) [34]. A 2022 retrospective study at a single center evaluated therapeutic hypothermia for HIE patients managed with either fentanyl or dexmedetomidine. Of the 45 patients, 19 were on fentanyl and 26 on dexmedetomidine. Patients on dexmedetomidine required fewer rescue boluses to achieve adequate sedation during therapeutic hypothermia compared to those on fentanyl. There were no significant differences in uncontrolled agitation scores or the need for additional agents. After rewarming, patients in the dexmedetomidine group had a reduced duration of sedation (0.52 days) compared to the fentanyl group (5 days) (*p* = 0.001). The dexmedetomidine group was also extubated earlier, at 3.1 days, and had earlier initiation of enteral feeds, at 8.5 days, compared to the fentanyl group, which had extubation at 11.3 days and feeding at 13 days (*p* = 0.004, 0.03). In terms of seizure events, the dexmedetomidine group had three cases, while the fentanyl group had seven, though this was not statistically significant (*p* = 0.07) [35]. This supports the notion that dexmedetomidine administration during therapeutic hypothermia for patients with HIE may be equally effective in controlling agitation and may minimize the need for additional medications. This could result in shorter durations of invasive ventilation and faster attainment of enteral feeding. Further studies are needed to evaluate dexmedetomidine’s role in seizure reduction and neuroprotection, in line with animal studies suggesting that dexmedetomidine may prevent neuroapoptosis induced by other agents [36].

With the growing body of literature on dexmedetomidine usage in neonates, including dose-escalation and pharmacokinetic studies [37], several drug formulary dosage guides have been developed by neonatology groups. These guides cover both intravenous and intranasal routes, with evidence derived from neonatal, pediatric, and adult studies [38]. The suggested dosage was adjusted for gestational age based on the available literature. Further research on varying levels of prematurity is required to optimize dosage ranges. The summary of intravenous dosage based on gestational age includes loading doses of 0.2–0.5 mcg/kg over 15 min, infusion doses of 0.2–0.75 mcg/kg/h, and a maximum dose of 1–1.5 mcg/kg/h. The recommended titration frequency is every 30 to 60 min. Additionally, intranasal dexmedetomidine may be administered 30 to 45 min before procedures at a dosage range of 0.5–4 mcg/kg [38].

The role of dexmedetomidine is expected to expand further with emerging strategies such as concurrent use with nerve blocks to minimize the need for operative endotracheal intubation. For example, a randomized controlled trial (RCT) involving 104 infants (75% preterm) who underwent elective bilateral inguinal hernia surgeries compared two approaches: 51 patients who received dexmedetomidine sedation combined with a localized caudal block anesthesia technique, and 46 patients who were managed with general sevoflurane anesthesia and invasive ventilation alongside a similar caudal block. The patients had a mean weight of 3.5 kg and a corresponding mean postmenstrual age of 41 weeks at the time of the operation. Among the dexmedetomidine cohort, 90.2% (n = 46) completed the surgery using this approach, while 5.9% (n = 3) were given a short course of sevoflurane at minimal dosage or nitrous oxide. Additionally, 3.9% (n = 2) required eventual invasive ventilation with general anesthesia intubation. In conclusion, with 96.1% avoiding invasive ventilation, this RCT suggests that this strategy offers a practical anesthesia approach for pediatric and neonatal hernia repair, although 9.8% may still require supplemental methods [39]. Another developing strategy is the use of dexmedetomidine for opioid withdrawal. Reports stemming from case and retrospective studies have included 20 pediatric subjects [40].

## 3. Striking the Balance

Although experience with dexmedetomidine in neonates is still limited compared to opioids and benzodiazepines, more evidence is needed to better evaluate its risks and side effects in this population.

Regarding pharmacokinetics, significant variability among individuals has been observed, with factors such as weight and liver function playing a significant role. Additional considerations include albumin levels in plasma and cardiac output. A pharmacokinetic study conducted in a neonatal and infant population with 20 subjects requiring invasive ventilation (median postmenstrual age: 44 weeks, range 33 to 61 weeks; weight: 2 to 6 kg) demonstrated that smaller gestational age was associated with reduced clearance capacity, which was statistically significant. The median maximum infusion dosage was 1.8 mcg/kg/h. Moreover, subjects with a history of heart surgery exhibited about 40% poorer clearance. The distribution volume was reported as 1.5 L/kg, with clearance predicted to range from 0.87 to 2.65 L/kg/h [41].

For premature infants, studies have indicated that this cohort may have reduced plasma clearance when adjusted for weight, with differences of 0.3 L/h/kg in preterm babies versus 0.9 L/h/kg in term infants. Premature infants also have a longer half-life for drug elimination—7.6 h compared to 3.2 h in term infants. Despite these differences, the preterm cohort has been able to achieve satisfactory sedation with dexmedetomidine alone, although similar infusion rates of around 0.2 mcg/kg/h may not be sufficient for most larger term infants [37]. Another pharmacokinetic study focused on clearance maturation involved 95 subjects ranging from 1 week to 14 years old, with weights ranging from 3 kg to 58.9 kg. At birth, term infants demonstrated a clearance rate of 18.2 L/h per 70 kg. By 1 year of age, this increased to 84.5% of the mature value. As noted elsewhere in the literature, this study also showed that patients who received dexmedetomidine infusions after heart surgery had around 27% less clearance compared to those who received a bolus dose [42,43]. Therefore, it would be prudent to place greater research emphasis on the pharmacokinetics and pharmacodynamics of dexmedetomidine, particularly in relation to prematurity, developmental changes in growing infants, and its relationship with cardiovascular conditions and procedures.

Caution is required when prescribing dexmedetomidine clinically in neonates and infants, given the differences in gestation, weight, underlying conditions, and the dynamic nature of a growing child, all of which contribute to variations in pharmacokinetics. One of the primary concerns with dexmedetomidine use is its potential for hemodynamic alterations. A study on healthy adult subjects using compartmental modeling reported that cardiac output could be reduced by 3% to 19%, even when the predicted medication levels were appropriate [44]. Hypotension may develop as a result of dexmedetomidine’s impact on parasympathetic efferent neuronal regulation of the heart [45]. Another concerning side effect is bradycardia, which has been reported in pediatric anesthesia studies with an occurrence rate of approximately 3% [46]. Newborns receiving dexmedetomidine for perioperative purposes may also face challenges with thermoregulation, as episodes of hypothermia have been reported [47], which may further exacerbate bradycardia. In one case, a term neonate weighing 3 kg, undergoing bladder exstrophy repair on day 2 of life, experienced clinically significant bradycardia during intraoperative hypothermia, necessitating a reduction in dexmedetomidine dosage [48]. Additionally, two pediatric patients with traumatic brain injury undergoing therapeutic hypothermia showed significant bradycardia when dexmedetomidine was added to their regimen [49]. A small case series on neonatal anesthesia suggested that bradycardic events may respond to atropine treatment [47].

Overall, the hemodynamic effects of dexmedetomidine appear to be predictable, dose-dependent, and reversible with either cessation or gradual weaning of the medication dose [37]. The administration of a loading dose or increased infusion rates may impact hemodynamic profiles, with these effects being typically distinct from those caused by other factors [50]. A retrospective observational case-control study compared fentanyl (n = 24) to dexmedetomidine (n = 24) in mechanically ventilated premature neonates with a mean birth weight of 832 g (±204.2 g) and gestational age of 25.5 weeks (±1.7 weeks). The study found no adverse events in the dexmedetomidine group, which received a loading dose of 0.5 mcg/kg followed by maintenance at 0.3 mcg/kg/h, with titrations of 0.1 mcg/kg/h. The mean dexmedetomidine infusion rate was 0.6 mcg/kg/h (range: 0.3–1.2 mcg/kg/h) [51]. This suggests that dexmedetomidine can be safely used in preterm neonates. However, the necessary precautions include careful monitoring for hemodynamic changes, bradycardia, and hypotension, as well as the cautious titration of dosages. In addition, formulary guidelines recommend avoiding additional intravenous bolus administration after the initial loading dose and stress the importance of controlled infusion rates to minimize hemodynamic instability [38].

Another important consideration is that prolonged use of dexmedetomidine may lead to withdrawal symptoms, particularly with sudden cessation of therapy. In children, withdrawal manifestations often include increased agitation, elevated blood pressure, and tachycardia [52,53,54,55]. Burbano et al. (2012) studied pediatric cardiac patients and found that 27% experienced agitation and tachycardia, while 35% experienced hypertension. These incidents were more prevalent in patients who had dexmedetomidine discontinued abruptly [52]. Further studies have indicated that approximately one-third of critically ill patients treated with dexmedetomidine for more than two days showed withdrawal symptoms [54,55]. To manage this, Liu et al. (2020) introduced a clonidine transition protocol for 22 infants and children (median age: 3.5 months, IQR 2–28.5 months). For patients on dexmedetomidine for more than three days, clonidine was initiated to minimize withdrawal effects. The managing team also had the liberty of commencing clonidine earlier on clinical grounds. While this research article did not provide detailed guidance on the weaning regimen for various age groups, it can be inferred that clonidine weaning typically begins 24 h after stopping dexmedetomidine. The study population was predominantly infants and children beyond the neonatal age group, with no preterm infants included [53]. Given that preterm infants have a slower clearance rate compared to term infants, resulting in a prolonged half-life of the medication [17], clinicians should exercise caution and use discretion when considering the weaning process, in addition to drug formulary guidelines [38]. The recommendation includes a slower weaning process for patients who have been on dexmedetomidine for 24 to 72 h. For dexmedetomidine use beyond 72 h, a clonidine transition protocol should be considered [38].

Table 1 provides the summary of key literature review done by the authors.

## 4. The New Paradigm

With the current evidence trend and motivation in achieving the desired equilibrium of patient comfort and minimizing short- and long-term side effects, the authors conclude that dexmedetomidine should be included in the consideration of neonatal sedation and analgesic strategies, especially when “conventional” agents have been provided in high doses or prolonged duration.

The authors recognize the limitations of the currently available literature on neonatal usage and advocate for the judicious use of dexmedetomidine. The authors emphasize that management should be tailored on a case-by-case basis, with particular focus on thorough hemodynamic assessment and controlled weaning after use for more than 24 h. Decision-making regarding dosage and duration should involve a combination of careful clinical evaluation and adherence to established dosing guidelines. Further research should explore specific circumstances, such as HIE with cooling therapy, given the potential neuroprotective benefits of dexmedetomidine.

## Figures and Tables

**Table 1:** Summary of key literature review.

Article	Description	Population	Dexmedetomidine Dosage	Beneficial Effects	Major Adverse Effects
He et al., 2013 [27]	Meta-analysis for 5 RCTs - Adenoidectomy or tonsillectomy - Dexmedetomidine vs. morphine and fentanyl	Pediatric Anesthesia	Not stated	Less respiratory depression	Lower analgesic effectiveness
Sun et al., 2014 [29]	Meta-analysis for 11 RCTs - Dexmedetomidine vs. midazolam	Pediatric Anesthesia	Not stated	- Better parent–child separation and mask compliance - Reduced need for breakthrough analgesia - Less delirium with agitation - Fewer shivering episodes after surgery	None
Chrysostomou et al., 2013 [30]	Retrospective care series - Dexmedetomidine for termination of reentrant supraventricular tachycardia	Neonates, Pediatric Cardiac Care	0.5–1.0 mcg/kg, slow intravenous push over 20 s	May decrease junctional ectopic tachycardia after congenital heart surgery	None
Curtis et al., 2023 [31]	Multicenter, observational cohort study - Dexmedetomidine vs. morphine and fentanyl in preterm infants	Neonates	Not stated	No difference compared to morphine and fentanyl	Minimal
Portelli et al., 2024 [33]	Meta-analysis for 6 studies - Dexmedetomidine Sedation and analgesia for mechanical ventilation or therapeutic hypothermia	Neonates	Loading dose: 0.05 to 0.5 mcg/kg, infusion 10 to 60 min Maintenance infusion dose: 0.05 to 1.2 mcg/kg/h	Effective in: - Providing analgesia and sedation - Minimizing the need for other medications - Reducing the time to extubate and shortening invasive ventilation requirements - Accelerating the attainment of enteral feeding	None
Surkov 2019 [34]	RCT - Dexmedetomidine vs. morphine in term babies with HIE receiving therapeutic hypothermia	Neonates	0.5 mcg/kg/h via continuous infusion	Less: - Inotropes dosage - Seizures - Negative neurological consequences	None
Naveed et al., 2022 [35]	Retrospective study, single center - Dexmedetomidine versus fentanyl late preterm and term babies with HIE receiving therapeutic hypothermia	Neonates	Mean initial dose of 0.16 ± 0.06 mcg/kg/h, with a maximum dose of 0.27 ± 0.12 mcg/kg/h	- Extubated earlier - Earlier initiation of enteral feeds	Treatment failure requiring transition to alternative sedatives
Gong et al., 2017 [46]	Meta-analysis for 21 studies - Dexmedetomidine anesthesia and incidence of bradycardia	Pediatric Anesthesia	Initial dose: 1.63 ± 0.33 mcg/kg Maintenance infusion dose: 0.86 ± 0.68 mcg/kg/h Total dose: 26.7 ± 20.8 mcg/kg	Not stated	Bradycardia incidence: 3%
Dilek et al., 2011 [47]	Prospective study - Dexmedetomidine and sevoflurane as general anesthesia for abdominal surgical procedures	Neonatal Anesthesia	Initial dose: 1 mcg/kg Maintenance infusion dose: 0.5 mcg/kg/h	Not stated	Hypothermia Bradycardia
O’Mara et al., 2012 [51]	Retrospective observational case-control study - Dexmedetomidine vs. fentanyl in mechanically ventilated premature neonates	Neonates	Loading dose: 0.5 mcg/kg Maintenance infusion dose: 0.3 mcg/kg/h, with titrations of 0.1 mcg/kg/h Mean infusion dose: 0.6 mcg/kg/h (range: 0.3–1.2 mcg/kg/h)	No difference compared to fentanyl	None
Burbano et al., 2012 [52]	Retrospective case series - Assessing dexmedetomidine withdrawal after usage for more than 3 days	Pediatric Cardiac Care	Mean infusion dose (Less than 1 years-old): 0.76 μg/kg/h Mean infusion dose (More than 1 years-old): 0.70 μg/kg/h	Not stated	Withdrawal features: - Agitation - Tachycardia - Hypertension
Liu et al., 2020 [53]	Retrospective, single-center study - Assessing dexmedetomidine withdrawal management with clonidine transition protocol	Pediatrics	Median infusion dose: 1.2 mcg/kg/h (IQR, 0.87–1.50) Median maximum dose: 1.65 mcg/kg/h (IQR, 1.4–2.0)	Not stated	Clonidine required to minimize withdrawal effects

## Abbreviations

The following abbreviations are used in this manuscript:

EUROPAIN	European Pain Audit In Neonates
NICUs	Neonatal intensive care units
PICU	Pediatric intensive care unit
RCTs	Randomized controlled trials
IQR	Interquartile range
HIE	Hypoxic-ischaemic encephalopathy
